# Retinoic acid protects human breast cancer cells against etoposide-induced apoptosis by NF-kappaB-dependent but cIAP2-independent mechanisms

**DOI:** 10.1186/1476-4598-9-15

**Published:** 2010-01-26

**Authors:** Ana M Jiménez-Lara, Ana Aranda, Hinrich Gronemeyer

**Affiliations:** 1 Instituto de Investigaciones Biomédicas de Madrid "Alberto Sols". CSIC/UAM. Arturo Duperier, 4. 28029 Madrid, Spain; 2 Institut de Génétique et de Biologie Moléculaire et Cellulaire, CNRS/INSERM/Université Louis Pasteur, BP 10142, 67404 Illkirch Cedex, France

## Abstract

**Background:**

Retinoids, through their cognate nuclear receptors, exert potent effects on cell growth, differentiation and apoptosis, and have significant promise for cancer therapy and chemoprevention. These ligands can determine the ultimate fate of target cells by stimulating or repressing gene expression directly, or indirectly through crosstalking with other signal transducers.

**Results:**

Using different breast cancer cell models, we show here that depending on the cellular context retinoids can signal either towards cell death or cell survival. Indeed, retinoids can induce the expression of pro-apoptotic (i.e. TRAIL, TNF-Related Apoptosis-Inducing Ligand, Apo2L/TNFSF10) and anti-apoptotic (i.e. cIAP2, inhibitor of apoptosis protein-2) genes. Promoter mapping, gel retardation and chromatin immunoprecipitation assays revealed that retinoids induce the expression of this gene mainly through crosstalk with NF-kappaB. Supporting this crosstalk, the activation of NF-kappaB by retinoids in T47D cells antagonizes the apoptosis triggered by the chemotherapeutic drugs etoposide, camptothecin or doxorubicin. Notably apoptosis induced by death ligands (i.e. TRAIL or antiFAS) is not antagonized by retinoids. That knockdown of cIAP2 expression by small interfering RNA does not alter the inhibition of etoposide-induced apoptosis by retinoids in T47D cells reveals that stimulation of cIAP2 expression is not the cause of their anti-apoptotic action. However, ectopic overexpression of a NF-kappaB repressor increases apoptosis by retinoids moderately and abrogates almost completely the retinoid-dependent inhibition of etoposide-induced apoptosis. Our data exclude cIAP2 and suggest that retinoids target other regulator(s) of the NF-kappaB signaling pathway to induce resistance to etoposide on certain breast cancer cells.

**Conclusions:**

This study shows an important role for the NF-kappaB pathway in retinoic acid signaling and retinoic acid-mediated resistance to cancer therapy-mediated apoptosis in breast cancer cells, independently of cIAP2. Our data support the use of NF-kappaB pathway activation as a marker for screening that will help to develop novel retinoids, or retinoid-based combination therapies with improved efficacy.

## Background

The search for alternatives to, and adjuvants for chemotherapy of breast cancer to prolong survival after the development of chemoresistance or during chemotherapy constitutes an area of intensive research. In this respect the concept of "cancer differentiation therapy" has emerged as an approach that intends to force a tumor cell to acquire a less aggressive differentiated phenotype, concomitant with growth inhibition and ultimately to induce cell death upon terminal differentiation. It has been reported that retinoids exert cell-differentiating effects in a variety of cancer cells including breast cancer. Retinoids, derivatives of vitamin A, are ligands of the retinoid receptor subclass of the nuclear receptor superfamily, which comprises three retinoic acid receptors (RARα, -β, and -γ) and three retinoid-X-receptors (RXRα, -β, and -γ) which form RAR/RXR heterodimers that are believed to correspond to the *in vivo *mediators of the ligand-induced signaling and regulate a plethora of direct and indirect gene regulatory programs [1]. Retinoids regulate important biological processes, such as embryo development, control and maintenance of organ homeostasis, and at the cellular level growth, differentiation and death [2,3]. These properties make retinoids promising agents in cancer therapy and chemoprevention [4]. Particularly, all-trans retinoic acid (atRA) is the prototypic "cancer differentiation therapy" of human acute promyelocytic leukemia (APL) that combined with anthracyclins cures 70-80% of patients [5]. Several groups have reported that retinoid analogs with agonistic or antagonistic activity can inhibit the growth [6-9], induce apoptosis [10,11] or cause differentiation of breast cancer cell lines [12,13]. Other groups have noted the capacity of retinoids to inhibit mammary carcinogenesis in animal models [14-16]. Previous studies suggest that retinoids inhibit cell growth interfering with growth factor signaling pathways [17,18].

The mammalian inhibitor of apoptosis proteins (IAPs), also known as baculovirus IAP repeat (BIR)-containing proteins (BIRCs), are evolutionary conserved proteins defined by their structural similarity. They share one to three copies of a well-conserved domain of about 70 aminoacids, named BIR. The first IAP was identified in baculovirus by its capacity to mediate host cell viability during infection [19,20]. Accordingly, members of this family particularly cellular IAPs (cIAP1 and cIAP2) and the X-chromosome-linked IAP (XIAP) have been shown to be able to protect or delay cell death in response to apoptotic stimuli when overexpressed [20]. IAPs have been demonstrated to inhibit cell death by directly repressing the proapoptotic activity of a family of cysteine proteases, caspases, as well as targeting proapoptotic components, such as Smac/DIABLO, for ubiquitin degradation [21-23]. IAP-deficient mice, although developing normally, revealed the importance of these proteins in survival, proliferation and some differentiation processes. Thus, NAIP, cIAP2 and XIAP have been shown to support survival of neurons [24], cardiomyocytes [25] or macrophages [26] under stress conditions. On the other hand, IAP proteins are highly expressed in many human malignancies and play a role in promoting tumorigenesis through inhibition of cell death and cooperation with other signaling pathways associated with malignancies [27,28]. As such, cIAP1/2 were originally identified as TNFR2-associated proteins [29]. Furthermore, cIAP1/2 and the closely related XIAP are targets of NF-κB signaling pathway [30-32]. The inducible transcription factor NF-κB plays an important role in numerous biological processes, such as proliferation and differentiation of many different systems, including neuronal cells, mammalian skin, myoblast, osteoclast, and the innate and adaptative immune systems [33-36]. Furthermore, NF-κB-deficient mice and cells suggest an important role for this transcription factor in cell survival [37,38] and sensitivity of cancers to chemotherapy [39,40]. Based on the observation that inhibition of the inducible transcription factor NF-κB, augments apoptosis mediated by TNF and other stimuli, it has long been claimed that upregulation of cIAP1/2, as NF-κB-target genes, is responsible for resistance to cell death induced by TNF and other stimuli [41].

Here, we report that retinoic acid-induced differentiation and apoptosis is accompanied by induction of pro-survival and pro-apoptotic gene expression programs in breast cancer cells. In studying the retinoid-activated survival gene programs we have put particular emphasis on the role of retinoid-induced NF-κB/cIAP2 signaling pathway(s) on the sensitivity of breast cancer cells towards chemotherapy. By comparing different breast cancer cell lines, we found that pretreatment with retinoic acid can antagonize chemotherapy-induced cell death in a cell- dependent manner, which correlates with the activation of NF-κB/cIAP2 signaling pathway(s). Our data exclude cIAP2 and suggest that other regulator(s) of the NF-κB signaling pathway are targeted by retinoic acid to confer resistance to chemotherapy-induced cell death.

## Results

### 9-*cis*-retinoic acid induces either differentiation or cell death in breast cancer cells in a cell-context dependent manner

**Figure 1:**
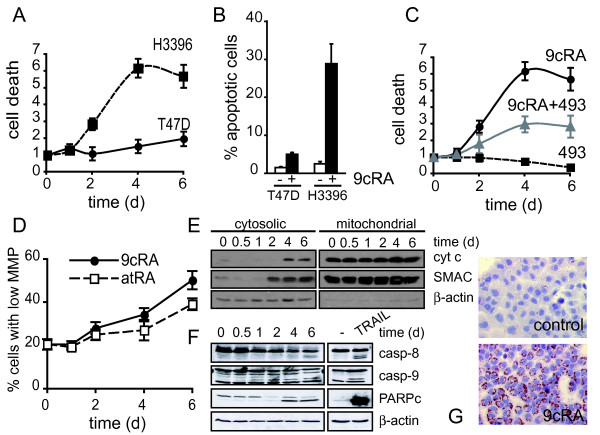
**Retinoic acid promotes either differentiation or cell death of breast cancer cells in a cell-context dependent manner**. (A) T47D and H3396 cells were treated with 9-*cis*-retinoic acid (9-cis-RA) for the indicated time and analyzed for the presence of DNA fragments in their cytosol as a measurement of cell death. Y-axis refers to the enrichment of histone complexed DNA fragments (mono- and oligonucleosomes) in the cytoplasm of apoptotic cells. The values represent the mean ± SD of three different experiments performed in triplicate. (B) T47D and H3396 cells were treated with 9-cis-RA for 6 days. Cell death was determined by FACS analysis after staining with propidium iodide as described in "Materials and Methods". The values represent the mean ± SD of three different experiments performed in duplicate. (C) H3396 cells were treated with 9-cis-RA and the RAR-antagonist BMS493, as indicated, and analyzed as described in (A). (D) H3396 cells were analyzed by flow cytometry to assess the population of cells with lower mitochondria membrane potential following all-*trans *retinoic acid (atRA) or 9-cis-RA treatment for the indicated time. The values represent the mean ± SD of three different experiments performed in duplicate. (E) Release of mitochondrial proteins to the cytosol was analyzed by western blotting after subcellular fractionation of H3396 cells treated with 9-cis-RA for the indicated time. (F) Effect of 9-cis-RA treatment on the cleavage of caspase-8 (casp-8), caspase-9 (casp-9) and PARP assessed by western blotting in H3396 cells. (G) Oil-red O staining of T47D cells untreated or treated with 1 μM 9-cis-RA for 72 h. The images shown are from one representative experiment performed three times with similar results.

It is well established that the inhibition of breast cancer cell proliferation by retinoids is accomplished by blocking cell cycle progression in the G1 phase. In order to find out whether there is a possible contribution of cell death to the antiproliferative effect of retinoids on breast cancer cells, we used a sensitive assay that measures the release of DNA fragments into the cytoplasm of cells. To maximally activate the RAR-RXR heterodimer, we used the pan-RAR and RXR agonist 9-*cis*-retinoic acid (9-cis-RA) to establish cell death kinetics. As shown in Fig. 1A-B, the treatment with 9-cis-RA at a pharmacological concentration of 10^-6 ^M is able to induce apoptosis in a cell context-specific manner. Indeed, while 9-cis-RA treatment does not significantly affect viability of T47D cells, it is able to induce apoptosis in the breast cancer cell line H3396. Induction of apoptosis by 9-cis-RA in this cell line requires RAR since treatment with a pan-RAR-antagonist, BMS493, blocks retinoid mediated apoptosis (Fig. 1C). That this block is partial may indicate a possible contribution of alternative rexinoid-induced death pathways which have been previously reported [42]. In these cells, mitochondrial membrane depolarization - a key event in apoptosis - is also induced by 9-cis-RA or by the RAR-pan-agonist all-*trans *retinoic acid (atRA). As shown in Fig. 1D, 9-cis-RA treatment clearly increases the number of cells presenting a diminished mitochondrial membrane potential in a time-dependent manner, and causes the release of the apoptogenic factors cytochrome c and SMAC/DIABLO from the mitochondria to the cytosol (Fig. 1E). Also, 9-cis-RA activates caspases 8 and 9 and the cleavage of a caspase-3 substrate, PARP, as assessed by western blot in H3396 cells. When H3396 cells were treated with TRAIL as positive control for the extrinsic death pathway, both caspase-8 and caspase-9 were activated and led to PARP cleavage (Fig. 1F). Together, these data show that retinoid-induced cell death in H3396 cells involves a crosstalk between the extrinsic and intrinsic death pathways.

In contrast to H3396, T47D cell growth was inhibited without loss of viability after 6 days of 1 μM 9-cis-RA treatment (Fig. 1A-B). Rather than inducing apoptosis, 9-cis-RA-treated T47D cells showed an increase in lipid droplet accumulation in the cytoplasm, demonstrated by Oil Red O staining and microscopic visualization, indicating differentiation of this cell line. T47D cells treated with 9-cis-RA look enlarged and the lipid droplets are disposed like a red perinuclear ring (Fig. 1G).

### 9-cis-RA induces the expression of cIAP2 in breast cancer cells in a cell context-dependent manner

**Figure 2:**
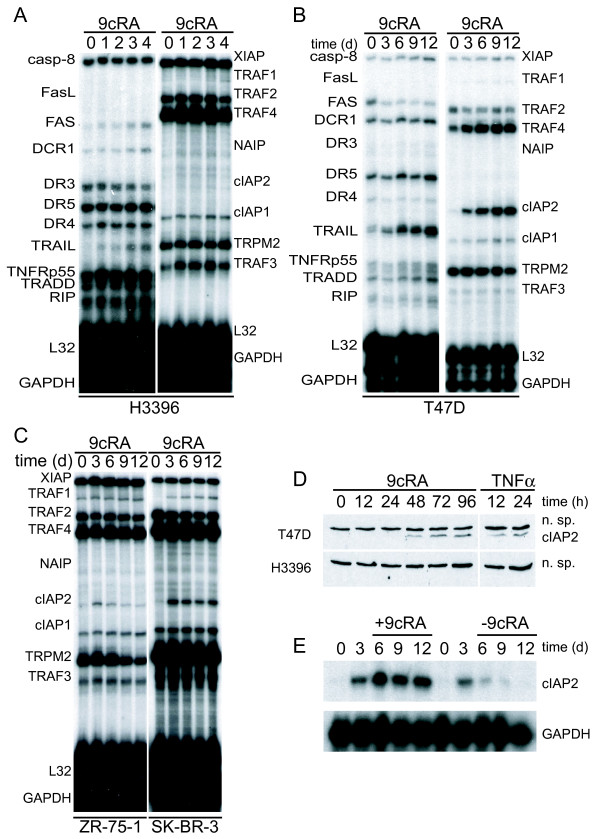
**9-cis-RA induces the expression of cIAP2 in breast cancer cells in a cell context dependent manner**. (A, B, C) Multiplex RNase protections assays (RPAs) to monitor the effect of 9-cis-RA on the expression of death receptor, death ligands, IAP and TRAF family members in four different breast cancer cell lines. Breast cancer cells were treated for the indicated time with 9-cis-RA at a concentration of 10^-6 ^M. (D) Western blot of whole cell extracts of 9-cis-RA-treated T47D cells and H3396 cells for 0, 12, 24, 48, 72 and 96 hours with anti-cIAP2. The nonspecific signal (n. sp.) confirms equal loading. As a positive control, breast cancer cells were treated with 50 μg/ml of hTNFα for 24 and 48 hours. (E) Reversibility of 9-cis-RA-induced cIAP2 gene expression. T47D cells were treated either in the absence or presence of 1 μM 9- cis-RA and after 3 days, total RNA was extracted. In parallel flasks, the medium was removed, and cells were washed and treated with either fresh control medium or medium with 10^-6 ^M of 9-cis-RA and grown for additional 3, 6 and 9 days. Media and ligands were renewed every 3 days. RNA was isolated and analyzed by RPA as described in (A). Equal loading was confirmed by GAPDH RNA level. The images shown are from one representative experiment performed twice with similar results.

In order to understand the mechanisms underlying the differential effects of retinoic acid on breast cancer cells, we treated several breast cancer cell lines for different times with 9-cis-RA and analyzed by RNase protection assay the gene expression levels of different key players in cell death and survival. Among the different proapoptotic genes analyzed, we observed significant upregulation of TRAIL and FAS mRNAs by 9-cis-RA in H3396 cells (Fig. 2A, left panel). In T47D cells, we did not observe a significant change of FAS but TRAIL messenger was upregulated at 6, 9 and 12 days (Fig. 2B, left panel). Additionally, we analyzed the expression of different members of the BCL2 family, as well as some members of the apoptosis-inhibitor proteins, IAPs. We did not observe significant changes in mRNA expression of the antiapoptotic BCL2 family members tested in either H3396 or T47D cells (data not shown). However, cIAP2, a known target of NF-κB, was strongly induced by 9-cis-RA in T47D but not in H3396 cells (Fig. 2A-B, right panels). We found that induction of cIAP2 gene expression by 9-cis-RA is not restricted to T47D cells, since 9-cis-RA was also able to induce cIAP2 in ZR-75-1 and SK-BR-3 breast cancer cell lines (Fig. 2C). At the protein level, 9-cis-RA induced cIAP2 in T47D but not in H3396 cells (Fig. 2D). Induction of cIAP2 gene expression is a reversible process, since removal of 9-cis-RA from cell culture media caused a time-dependent reduction of cIAP2 gene expression, reaching near-basal levels after 9 days (Fig. 2E). All together, these data show that 9-cis-RA can induce in a cell context-dependent manner pro-survival and pro-apoptotic gene programs in breast cancer cells.

### 9-cis-RA activates cIAP2 transcription through NF-κB response elements and induces *in vivo *recruitment of p65 and RAR to the cIAP2 promoter

**Figure 3:**
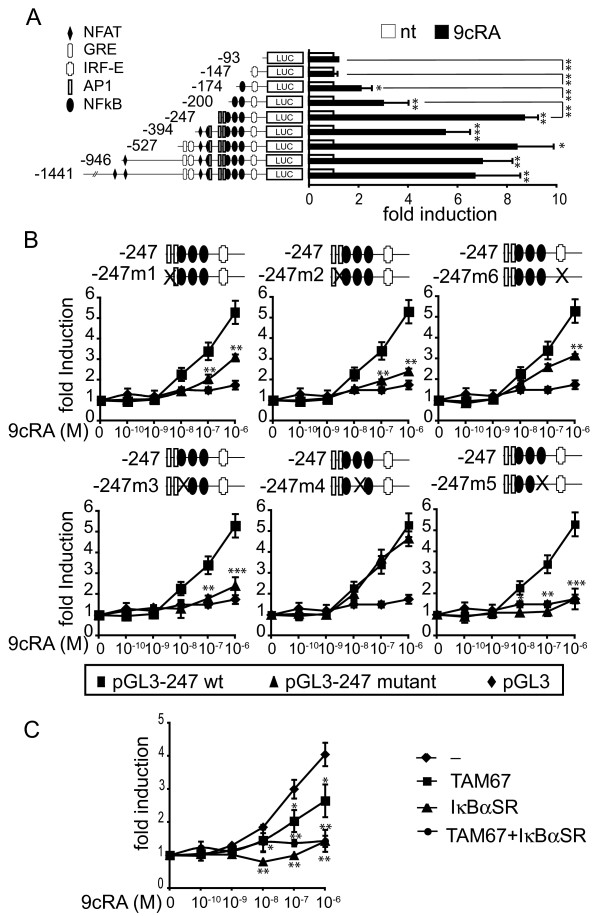
**9-cis-RA activates cIAP2 transcription through NF-κB response elements**. (A) Illustration of the reporter constructs containing 5'-deletion fragments of the cIAP2 upstream regulatory region. Luciferase activity in SK-BR-3 cells transiently transfected with the indicated cIAP2 reporter gene in the presence (black bars) and absence (white bars) of 9-cis-RA. The data shown represent the mean ± SD of three independent experiments performed in duplicate. The luciferase levels were normalized with those of β-galactosidase and expressed as the induction over the controls. (B) Transfection experiments as described in (A) but using site-specific mutants derived from -247-cIAP2 promoter as depicted (Black triangle), the wild-type promoter (Black square) and the backbone vector pGL3 (Black diamond). After transfection, SK-BR-3 cells were treated with different doses of 9-cis-RA, as indicated. The data shown represent the mean ± SD of three independent experiments performed in duplicate. The luciferase levels were normalized with those of β-galactosidase and expressed as the induction over the controls. Asterisks denote the existence of statistically significant differences between the wild-type and mutant promoter constructs. (C) SK-BR-3 cells were transiently co-transfected with the -247- cIAP2 reporter gene and pSG5, pSG5-IκBαSR(S32A/S36A) or pcDNA-TAM54 (a dominant-negative version of c-JUN) and either untreated or treated with 9-cis-RA, as described in (B). The luciferase levels were normalized with those of β-galactosidase and expressed as the induction over the controls. The values represent the mean ± SD of three experiments performed in duplicate. Asterisks denote statistically significant differences against cells transfected with an empty vector.

Transient transfection in SK-BR-3 cells of a chimeric luciferase reporter gene driven by the cIAP2 promoter demonstrated that a 1.4-kilobase sequence upstream of the transcription initiation site contains retinoic acid-inducible elements. Previously the presence of a glucocorticoid response element (at position -514), four Nuclear Factor of Activated T cells (NFAT) binding sites (at positions -1086, -821, -354 and -301), three potential binding sites for Activator Protein-1 (AP1) (at positions -294, -233 and -220), two Interferon Response Elements (IRF-E) (at positions -475 and -130) and three NF-κB binding sites (at positions -210, -197 and -147) in the proximal promoter of the cIAP2 gene have been predicted [31] but sequence analysis did not show the presence of consensus retinoic-acid response elements (RAREs) that could mediate stimulation by 9-cis-RA (Fig. 3A). Promoter mapping initially narrowed the retinoic acid responsive sequence down to 174 base pairs (bp), which in addition to the TATA box, contains an Interferon Response Element and the NF-κB site 3. This promoter fragment showed a 2-fold 9-cis-RA inducibility, similar to that found with -200-cIAP2-Luc construct, which contains an additional NF-κB site. However, the induction by 9-cis-RA of the -247-cIAP2-Luc construct, which contains three NF-κB sites and two AP1 sites, was significantly higher (about 9-fold), suggesting a potential role of (some of) these elements in the stimulation by the retinoid. No significant variation was seen when 9-cis-RA-induced transcriptional activity between -247-cIAP2-Luc and longer cIAP2 promoter constructs was compared. Because no obvious RAREs could be found in the 247 bp retinoic acid responsive sequence, we systematically mutated each putative cis-acting element in the background of -247-cIAP2-Luc to test if one of these response elements could mediate this response (Fig. 3B). Site directed mutagenesis of these response elements showed the critical importance of two NF-κB binding sites at positions -210 and -147 (m3 and m5 mutants, respectively) and one potential AP1 binding site at position -220, partially overlapping with the NF-κB binding site-1 (m2 mutant), and highlighted the contribution of the AP1 binding site at position -233 (m1 mutant) and the IRF-E site at position -130 (m6 mutant) to retinoid-induced promoter activity (Fig. 3B). To further reinforce these data, SK-BR-3 cells were transiently co-transfected with the -247-cIAP2 reporter gene and either an expression vector coding for a dominant negative mutant of IκBα, IκBα-SR(S32A/S36A) or an expression vector coding for a dominant negative of c-JUN (TAM-67), to test whether 9-cis-RA inducibility was impaired. Expression of the dominant negative mutant of IκBα totally blocked retinoid inducibility of the cIAP2 promoter, whereas expression of TAM-67 only partially suppressed retinoid-induced cIAP2 promoter activity, thus confirming the critical role of NF-κB in the induction of cIAP2 expression by 9-cis-RA (Fig. 3C). Together, these data clearly demonstrate that NF-κB is critically involved in mediating the retinoic acid-dependent transcriptional activation of the cIAP2 promoter and that potentially other factors, particularly c-JUN and IRFs, contribute to the overall response.

**Figure 4:**
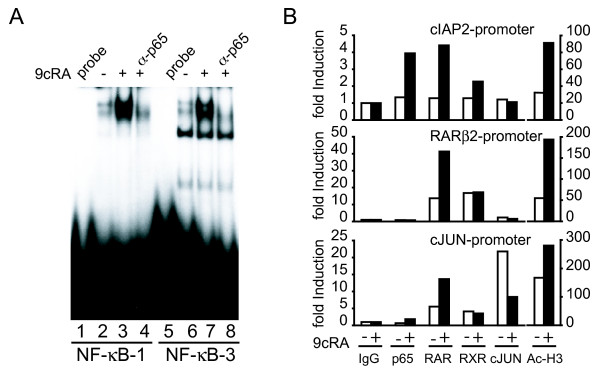
**9-cis-RA treatment results in *in vivo *recruitment of p65 and retinoic-acid receptors, RAR and RXR to the cIAP2 promoter**. (A) EMSA performed with probes bearing the NF-κB-1 and NF-κB-3 sites of the cIAP2 promoter and nuclear extracts from T47D cells treated with 1 μM 9-cis-RA for 1 h. When indicated, antibodies directed against p65 were present in the binding assays. The images shown are from one representative experiment performed twice with similar results. B) T47D breast cancer cells were treated as indicated in Material and Methods and ChIPs assays were performed using antibodies against p65, RARs, RXRα, cJUN or acetyl-H3 histone. Immunoprecipitated chromatin was analyzed by real time PCR using primers specific for the cIAP2, RARβ2 and cJUN promoters. Shown are data from a representative experiment, expressed as fold induction relative to the values obtained with immunoglobulin G (IgG) used as a negative control, which were set to 1. This experiment was repeated three times with similar results.

Since mutations of NF-κB binding sites resulted in a major decrease of 9-cis-RA inducibility, we tested these sites in electrophoretic mobility shift assays. EMSAs with extracts of T47D cells demonstrated that 9-cis-RA induces the binding of a protein complex to the cIAP2 NF-κB1 and NF-κB3 sites. Incubation with antibodies against p65 inhibited binding, revealing the presence of this NF-κB family member in these complexes (Fig. 4A). Therefore, we conclude that 9-cis-RA induces the formation of p65-containing complexes at the NF-κB binding sites of the cIAP2 promoter.

To gain a deeper insight into the molecular mechanisms underlying 9-cis-RA induction of cIAP2 transcription, we performed chromatin immunoprecipitation (ChIP) assays to assess the *in vivo *recruitment of p65, RAR, RXRα and c-JUN to the cIAP2 promoter in untreated and 9-cis-RA-treated T47D cells. ChIP assays revealed that 9-cis-RA induced acetylation of histone H3 at the cIAP2 promoter, a hallmark of transcriptional activation. In addition, we could not detect basal occupancy of the cIAP2 promoter by p65, RAR or RXRα, but significant occupancy of the promoter by these transcription factors was observed after exposure of T47D cells to 9-cis-RA. In contrast with the results obtained with the cIAP2 promoter, p65 was not recruited to the RARβ gene promoter, a well-characterized retinoic acid-responsive gene, where we were able to detect basal and induced recruitment of RAR and RXRα (Fig. 4B, middle panel). Whereas binding of cJUN to the cIAP2 promoter in 9-cis-treated T47D chromatin extracts was not observed (Fig. 4B, upper panel), strong occupancy of the cJUN proximal promoter, used as a positive control, was easily detected (Fig. 4B, lower panel). Interestingly, this binding was reduced in 9-cis-RA-treated cells. Together, these data suggest that the recruitment of NF-κB factors and retinoic-acid receptors might be responsible for 9-cis-RA induction of cIAP2 gene transcription.

### 9-cis-RA pretreatment prevents apoptosis induced by chemotherapy drugs in T47D cells: correlation with the activation of NF-κB/cIAP2 signaling pathway(s)

**Figure 5:**
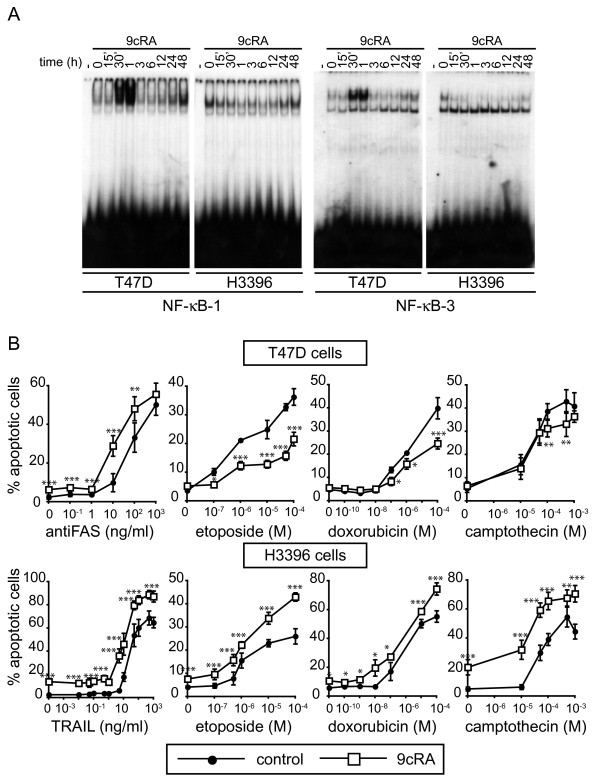
**9-cis-RA pretreatment prevents apoptosis induced by cancer chemotherapy insults in T47D cells, but increases chemotherapy-induced apoptosis in H3396 cells: correlation with NF-κB activity induction by 9-cis-RA**. (A) EMSA performed with probes bearing the NF-κB-1 and NF-κB-3 sites of the cIAP2 promoter and nuclear extracts from T47D and H3396 cells treated with 1 μM 9-cis-RA for different times, as indicated. The images shown are from one representative experiment performed twice with similar results. (B) T47D and H3396 cells were pretreated with or without 9-cis-RA for 30 h, followed by treatment with different doses of the indicated chemotherapeutic agents for 72 h and death ligands for 48 h. Apoptotic cells were determined by FACS analysis after staining with propidium iodide as described in "Materials and Methods". The values represent the mean ± SD of three independent experiments performed in duplicate. Asterisks denote statistically significant differences between untreated cells and cells treated with 9-cis-RA.

To explore whether cIAP2 induction may play a pro-survival role in T47D cells, we assessed the sensitivity of T47D and H3396 breast cancer cells pretreated with 9-cis-RA to a diverse set of death ligands and chemotherapy drugs. We reasoned that the induction of cIAP2 by 9-cis-RA could account for a decreased sensitivity of T47D cells to these compounds. On the other hand, we could anticipate that in H3396 cells, a cell-context where 9-cis-RA did not induce cIAP2, we would not find a decrease in the sensitivity to these drugs. First, we investigated NF-κB activation by 9-cis-RA in both breast cancer cells systems to analyze whether the absence of induction of cIAP2 expression in H3396 cells could be due to a defect in the ability of retinoids to activate NF-κB signaling in these cells. To provide evidence for this notion, we first performed EMSAs with nuclear protein extracts from T47D cells treated with 9-cis-RA for different time periods. 9-cis-RA induced the binding of protein complexes to the cIAP2 NF-κB binding sites-1 and -3 in a biphasic dynamics: a strong rise in NF-κB activation is observed between 30 min and 1 h after 9-cis-RA treatment, followed by another increment in NF-κB activation around 24-48 hours, although the latter appears to show a much weaker binding intensity. Parallel experiments in H3396 cells showed that 9-cis-RA did not induce the binding of protein complexes to the NF-κB sites at any time tested (Fig. 5A). These data suggested that the lack of induction of cIAP2 expression by retinoids in H3396 cells might indeed be due to a defect in the activation of NF-κB in these cells.

Death of T47D and H3396 cells, in the absence or presence of 9-cis-RA pretreatment, was examined after exposure to various apoptogenic insults: anti-FAS, TRAIL, etoposide, doxorubicin and camptothecin. As observed above, the treatment with 9-cis-RA alone did not affect viability of T47D cells. However, 9-cis-RA pretreatment decreased sensitivity of T47D cells to doxorubicin, etoposide and camptothecin (Fig. 5B), suggesting that the activation of NF-κB/cIAP2 signaling pathway(s) by retinoids in these cells correlates with an increase in apoptosis resistance. On the other hand, in H3396 cells where 9-cis-RA induces neither NF-κB activation nor cIAP2 expression but makes the cells enter a fully apoptotic program, death curves showed that the treatment with 9-cis-RA not only induced apoptosis by itself, but also increased in an additive manner the apoptosis in response to TRAIL, etoposide, doxorubicin or camptothecin (Fig. 5B). Note that, 9-cis-RA treatment augmented apoptosis mediated by the death receptor pathway in both cell lines. These results reveal that the activation of NF-κB/cIAP2 signaling pathway(s) by retinoids in a given breast cancer cell apparently correlates with the ability of these retinoids to protect cells against chemotherapy-induced apoptosis.

We further investigated the effect of 9-cis-RA in preventing etoposide-mediated apoptosis in one additional breast cancer cell line, ZR-75-1, where the retinoid upregulates cIAP2 expression and potentially NF-κB activation (Fig. 2C, left panel). ZR-75-1 cells responded to 9-cis-RA in a similar manner to T47D cells showing a reduction in sensitivity to etoposide upon pretreatment with 9-cis-RA (see Additional file 1). These results show that the protection against etoposide-mediated cell death exerted by 9-cis-RA is not restricted to T47D breast cancer cells.

### cIAP2 is not critically involved in the protection of etoposide-induced apoptosis by 9-cis-RA

**Figure 6:**
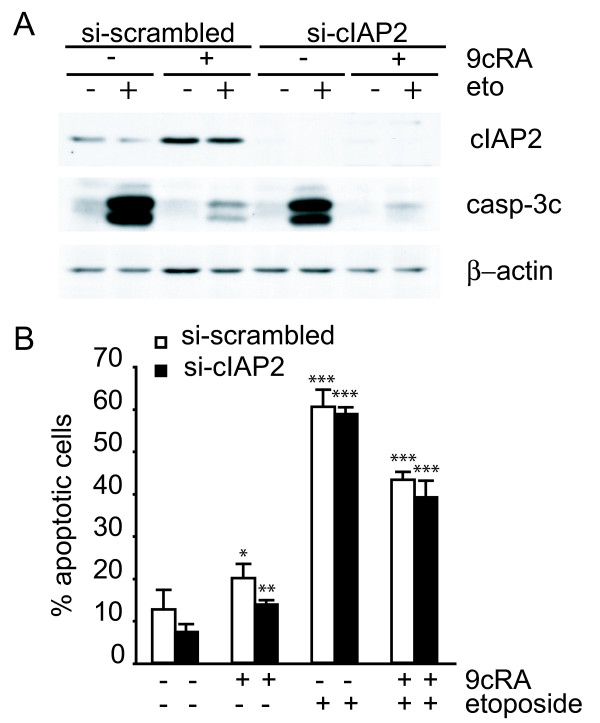
**Suppression of cIAP2 expression is not sufficient to abrogate 9-cis-RA inhibition of etoposide-induced apoptosis in T47D cells**. (A) T47D cells were transfected with either scrambled-siRNA or cIAP2-siRNA and pretreated with or without 9-cis-RA for 30 h, followed by treatment with etoposide 100 μM for 24 h. Cell lysates were analyzed by western blot for the expression of cleaved caspase-3, cIAP2 and β-actin using specific antibodies. The images shown are from one representative experiment performed three times with similar results. (B) T47D cells were transfected with either scrambled-siRNA (white bars) or cIAP2-siRNA (black bars). After 24 h, lipid-siRNA complexes were removed from media and cells were pretreated with or without 1 μM 9-cis-RA for 30 h, followed by treatment with etoposide 100 μM for 72 h. The percentage of apoptotic cells was determined by FACS analysis after staining with propidium iodide. The values represent the mean ± SD of three experiments performed in duplicate. Asterisks denote statistically significant differences against the corresponding untreated cells.

Previous reports have shown that exogenous overexpression of cIAP2 can abrogate apoptosis induced by genotoxic anticancer drugs such as etoposide, but not death receptor-mediated apoptosis in a NF-κB null background [41]. We have observed that 9-cis-RA protection against etoposide-mediated cell death correlates with cIAP2-induced expression by 9-cis-RA in T47D cells. To test whether cIAP2 has a role in this protection, expression of cIAP2 was suppressed by using transiently expressed siRNA. Although the cIAP2-siRNA efficiently downregulated both basal and 9-cis-RA-induced cIAP2 protein levels when compared to a scrambled siRNA (Fig. 6A), downregulation of cIAP2 was not sufficient to restore sensitivity to etoposide-mediated cell death in 9-cis-RA pretreated T47D cells (Fig. 6B). To further confirm the above results, we compared the level of activation of caspase-3 by western blot, as a measurement of cell death, between T47D cells transfected with a scrambled siRNA and T47D cells transfected with a siRNA against cIAP2. While the levels of cleaved caspase-3 were induced by etoposide and strikingly abrogated when cells were pretreated with 9-cis-RA in scrambled-siRNA transfected cells, downregulation of cIAP2 protein level did not restore the levels of cleaved caspase-3 induced by etoposide in the presence of 9-cis-RA (Fig. 6A). This reveals that cIAP2 is not critically involved in the inhibition of etoposide-induced apoptosis in 9-cis-RA pretreated T47D cells.

### Over-expression of the super-repressor of NF-κB activation, IκBα-SR(S32A/S36A), abrogates protection of etoposide induced apoptosis by 9-cis-RA

**Figure 7:**
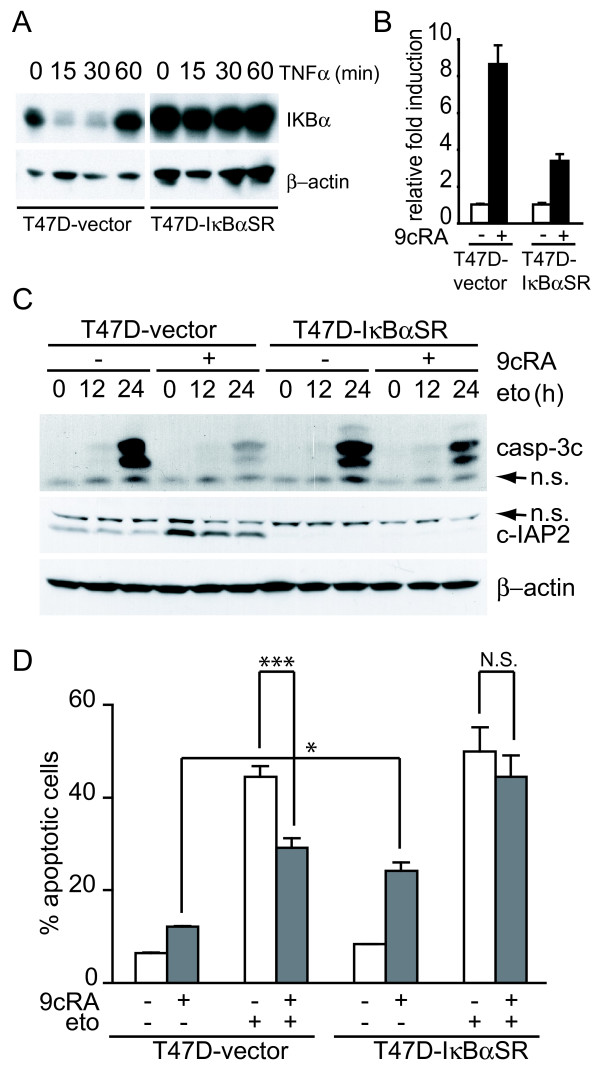
**Over-expression of the super-repressor of NF-κB activation, IκBα-SR(S32A/S36A), leads to significant abrogation of retinoic acid-mediated inhibition of etoposide-induced apoptosis**. (A) T47D-vector and T47D-IκBαSR cells were untreated or incubated for the indicated time with hTNFα (50 ng/ml). Extracts were analyzed by western blotting with an IκBα antibody. Equal loading was confirmed with an anti-β-actin antibody. The images shown are from one representative experiment performed three times with similar results. (B) T47D-vector or T47D-IκBαSR cells were untreated or treated with 9-cis-RA for 48 h and expression of cIAP2 and β-actin were analysed by Reverse-Transcriptase Polymerase Reaction and real time PCR. The values represent the mean ± SD of three different experiments performed in duplicate. (C) T47D-vector or T47D-IκBαSR cells were pretreated with or without 9-cis-RA for 30 h, followed by treatment with etoposide 100 μM as previously indicated. At the indicated times, cell lysates were analyzed by western blot for the expression of cleaved caspase-3, cIAP2 and β-actin using specific antibodies. The images shown are from one representative experiment performed three times with similar results. (D) T47D-vector or T47D-IκBαSR cells were pretreated with or without 9-cis-RA for 30 h, followed by treatment with etoposide 100 μM for 72 h. The percentage of apoptotic cells was determined by FACS analysis after staining with propidium iodide. The values represent the mean ± SD of three independent experiments performed in duplicate. Asterisks denote the existence of statistically significant differences between the indicated groups; N.S.: not significant (Student's t-test).

To explore whether NF-κB activation may play a role in protection of etoposide-induced apoptosis by 9-cis-RA, we generated T47D cells stably overexpressing a constitutively active form of IκBα, IκBα-SR(S32A/S36A). This mutated version of IκBα contains serine to alanine mutations at residues 32 and 36, which confer resistance to signal-induced phosphorylation and subsequent proteasome-mediated degradation. Thus, NF-κB dimers remain bound to IκBα-SR(S32A/S36A) in the cytosol, and their translocation to the nucleus and the subsequent transcriptional regulation of their target genes is impaired. The cDNA coding for IκBα-SR(S32A/S36A) was inserted into de pcDNA3.1 vector and after transfection in T47D cells, a pool of neomycin-resistant cells was isolated (T47D-IκBαSR cells). To determine the effectiveness of the super-repressor in the stable cell line, we assessed by western blotting IκBα resistance to TNFα-induced degradation. For this purpose, whole cell extracts were prepared from T47D-IκBαSR cells and control cells i.e. a pool of neomycin resistant cells isolated after transfection of the pcDNA3.1 vector (T47D-vector cells). As expected, IκBα was degraded after 15 min treatment with TNFα in T47D-vector cells, while degradation of IκBα was not affected by TNFα treatment in the case of T47D-IκBαSR cells (Fig. 7A). Furthermore, the expression of IκBα-SR(S32A/S36A) had no effect on proliferation rate of the stable cell line in the absence of treatment (not shown). Therefore, T47D-IκBαSR cells are a good tool to determine the involvement of the NF-κB pathway in the protection of apoptosis by 9-cis-RA.

As a further control of the efficiency of NF-κB inactivation, we evaluated both the basal and 9-cis-RA-induced level of the NF-κB-dependent cIAP2 mRNA and protein in the IκBα mutant cell line by real time PCR and western blot, and found that cIAP2 levels were specifically down-regulated when compared to control cells (Fig. 7B-C). To evaluate the impact of IκBαSR(S32A/S36A) overexpression in 9-cis-RA protection against etoposide-mediated apoptosis, we compared by Western blotting, as a measurement of cell death, the level of activation of caspase-3 between T47D-vector cells and T47D-IκBαSR cells. While the level of cleaved caspase-3 was induced by etoposide in control cells and strongly abrogated when cells were pretreated with 9-cis-RA, overexpression of the IκBα mutant did not affect notably caspase-3 activation by etoposide, but restored very significantly the activation of cleaved caspase-3 by etoposide in the presence of 9-cis-RA (Fig. 7C).

We also compared the apoptosis induced by etoposide in the presence or absence of 9-cis-RA pretreatment in T47D-vector and T47D-IκBαSR cells by propidium iodide staining and FACS analysis. As seen in Fig. 7D, while pretreatment with 9-cis-RA inhibits etoposide-mediated cell death in T47D-vector cells, apoptosis was enhanced in T47D-IκBαSR cells treated with 9-cis-RA alone, and these cells were equally sensitive to etoposide in the presence and absence of 9-cis-RA, showing again the important role of the NF-κB pathway in the protection of 9-cis-RA against apoptosis. These data strongly support that this protection is mediated by NF-κB dependent mechanisms.

## Discussion

A complex and intricate network of signaling pathways determines whether a cell will either proliferate, differentiate, survive or die. Retinoids, due to their strong differentiative potential, have been widely used for both cancer therapy and cancer prevention [43]. There are many examples in the literature of distinct cell types whose differentiation is under the control of retinoids: embryonal carcinoma cells, promyelocytic leukemia cells, neuroblastoma cells, normal erythroid progenitors, etc. [3,44-48]. In addition to differentiation induction, retinoids are able to initiate several other programs that may contribute to its therapeutical potential. Indeed, it has been shown that retinoids induce apoptosis of APL cells and blasts of APL patients through selective paracrine action of the death ligand TRAIL [49]. In breast cancer cells, we provide evidence that retinoic acid induces cell growth inhibition and depending on cell-context, promotes a sort of differentiation without affecting viability or makes the cells enter a fully apoptotic program. The finding that 9-cis-RA causes differentiation of T47D cells is in agreement with the previously reported accumulation of lipid droplets in cytoplasmic vesicles [50] and milk protein casein [51] in normal mammary epithelial cells, and in the breast cancer cell lines MCF7 and AU565 [52] treated with retinoids. However, further studies are needed to determine whether the differentiation characterized by accumulation of cellular lipid depots contributes to the antiproliferative effects of retinoic acid in breast cancer cells.

A circuitry of several apoptotic programs is induced in breast cancer cells by retinoic acid. We have previously provided evidence that retinoids promote the induction of TRAIL not only in hematopoietic but also in breast cancer cells [53]. In the current study, we have shown that induction of TRAIL and FAS by retinoic acid in the breast cancer cell line H3396 correlates with an increase in the number of apoptotic cells. In accordance with studies that report that TRAIL and FAS signal through caspase-8 activation, the activity of this enzyme is induced in H3396 cells treated with 9-cis-RA or with exogenous TRAIL. Although additional studies will be required to clarify the possible involvement of the extrinsic death pathway in retinoic-induced apoptosis in H3396 cells, activation of downstream caspases like caspase-9, as well as the release of cytochrome c and SMAC/DIABLO from the mitochondria to the cytosol and the loss of the mitochondrial membrane potential prove that the intrinsic pathway is dominantly involved in retinoic acid-induced apoptosis.

Paradoxically in certain breast cancer cells, retinoic acid induces concomitantly to TRAIL upregulation, the activation of a gene program of apparently opposite functionality, characterized by the induction of the antiapoptotic IAP family member, cIAP2, a NF-κB target gene. cIAP2 expression was significantly modulated at the mRNA and protein levels by retinoic acid in a cell context dependent manner. Using promoter mapping, promoter site-directed mutagenesis, EMSAs and chromatin immunoprecipitation analysis we show that retinoic acid induces the recruitment of NF-κB proteins to NF-κB binding sites in the proximal region of the cIAP2 promoter, thereby causing induction of cIAP2 expression. In agreement with our data, the induction of NF-κB proteins binding and activity by retinoic acid has been reported in several cell systems such as neuroblastoma or leukemia cells [49,54,55]. Importantly, in addition to NF-κB proteins, the retinoid receptors, RAR and RXR, are also recruited *in vivo *to the cIAP2 promoter upon retinoic acid treatment, despite the absence of *bona fide *RARE sites in this promoter by *in silico *analysis. Protein-protein interaction between p50/p65 and RXR that could contribute to stabilize the transcriptional activation complex have been described [56]. Despite the finding that mutation of an AP-1 motif decreases 9-cis-RA inducibility, we could not detect *in vivo *recruitment of cJUN to the cIAP2 promoter in response to the retinoid. Although we cannot totally dismiss the possibility that cJUN takes part of the transcriptional complex induced by retinoic acid, other AP-1 binding factors could be recruited to the promoter. Importantly, although our data suggest that ligand-bound RAR/RXR may be recruited directly to the transcriptional activation complex we cannot discard that, in addition, retinoic acid induction of cIAP2 expression proceeds via regulatory circuits, which are likely to involve retinoic acid-target genes as well as cross-talk with other signaling pathways. Thus, as reported for neuroblastoma cells [57], retinoic acid could induce the activation in breast cancer cells of the phosphatidylinositol 3-Kinase/Akt signaling pathway that finally results in NF-κB activation.

Little is known about the anti-apoptotic potential of retinoic acid [58-61]. We provide evidence that in a cellular context, present in T47D, ZR-75-1 and SK-BR-3 cells, retinoic acid markedly upregulates cIAP2 expression. Retinoic acid significantly mitigates the apoptosis induced by chemotherapeutic agents in T47D and ZR-75-1 cells, while it is able to increase apoptosis by these compounds in H3396 cells where retinoic acid does not induce cIAP2 expression. Many antiapoptotic proteins, such as Bcl-2, Mcl-1 and Bcl-XL, have been shown to inhibit chemotherapeutic agent-induced apoptosis in diverse cell system models including hematopoietic and neuroblastoma cells. Additionally, it has been shown that the activation of genes encoding TRAF and IAP proteins by NF-κB serves to block apoptosis promoted by different insults including chemotherapy-induced apoptosis in different cell types [30,32,39,62]. In particular, overexpression of cIAP2 inhibits etoposide-induced apoptosis, processing of caspase-3 and generation of caspase-like protease activity in 293T cells [21]. Accordingly, it has also been shown that cIAP2 overexpression blocked etoposide-induced processing of caspase-3 and apoptosis in HT1080 cells under NF-κB-null conditions [41]. Thus, cIAP2 emerged as a likely candidate to mediate the antiapoptotic effect of retinoic acid in our cell system. To test the involvement of cIAP2 in retinoic acid action, we performed siRNA studies to selectively suppress cIAP2 expression. Notably however, these studies did not show sensitization of T47D cells to etoposide-induced apoptosis in conditions of retinoic acid pretreatment, despite effective cIAP2 downregulation. These findings clearly demonstrated that cIAP2 is not necessary for retinoic acid protection of chemotherapy-induced apoptosis. However, we cannot rule out the possibility that compensatory expression of other members of the IAP family protein could supersede the absence of cIAP2 in our system, explaining the lack of effect of cIAP2 knockdown. Recent data also suggest that neither cIAP1 nor cIAP2 are able to inhibit caspases directly [63]. Thus, these results and ours suggest a more complex role for cIAP2 in antiapoptosis than previously expected. Further studies are required to reveal the precise involvement of cIAP2 on retinoic acid effects in breast cancer cells.

It has been reported that the NF-κB signaling pathway plays a major role in cell survival [64] and in sensitivity of cancers to chemotherapy [40]. In accordance with these observations, we have found that retinoic acid can activate the NF-κB signaling pathway in certain breast cancer cells, which correlates with the induction of resistance against apoptosis induced by cancer therapy agents, such as etoposide, doxorubicin or camptothecin. Furthermore, we have demonstrated that impairment of NF-κB activation results in a moderate increment of retinoic acid-induced apoptosis and in a similar sensitivity to etoposide in the presence and absence of 9-cis-RA. The multiplicity of mechanisms whereby NF-κB serves the antiapoptotic function is becoming increasingly complex. It has been reported that NF-κB increases the expression of several antioxidant effectors, such as glutathione cysteine synthetase, glutathione, manganese superoxide dismutase, hemeoxygenase, ferritin heavy chain and thioredoxin [65-69]. On the other hand, retinoic acid has been shown to reduce susceptibility to oxidative stress in chick embryonic neurons [70], in PC12 cells, and in mesangial cells [58], although the mechanism of the antioxidant effect of retinoic acid remains unclear. Furthermore, it has been reported that retinoic acid treatment represses ROS accumulation by a mechanism involving NF-κB in NB4 cells; in these studies, the impairment of NF-κB activation resulted in increased ROS levels and JNK activation in retinoic treated NB4 cells [55]. Since etoposide induces marked biochemical alterations characteristic of oxidative stress, including enhanced lipid peroxidation and decreased levels of reduced glutathione, it will be of interest to determine the role of different antioxidant effectors in retinoic acid protection of etoposide-induced apoptosis. It is tempting to speculate that retinoic acid is able to regulate the sensitivity to chemotherapeutic agents-induced apoptosis by increasing antioxidant defense components through NF-κB proteins in certain cellular contexts such as T47D breast cancer cells.

## Conclusions

This study illustrates the multiplicity of pathways induced by retinoids in breast cancer cells that can cause markedly different responses depending on the specific cellular context: retinoids can signal towards cell death or cell survival. Moreover, the results of this study support an important role for the NF-κB pathway in retinoic acid signaling and retinoic acid-mediated resistance to cancer therapy-mediated apoptosis in breast cancer cells, independently of cIAP2. Our data support the use of NF-κB pathway activation as a marker for screening that will help to develop novel retinoids, or retinoid-based combination therapies with improved efficacy. Additionally, this study further validates current efforts aimed to inhibit NF-κB signaling pathways to improve clinical therapies.

## Methods

### Cell culture and treatment

H3396, T47D, ZR75-1 cells were cultured in RPMI or Dulbecco in the case of SK-BR-3 cells, containing red phenol with 10% foetal calf serum, 100 U/ml penicillin, 100 U/ml streptomycin, and 2 mM glutamine. For the T47D cell line, medium was supplemented with 0,6 μg/ml insulin. 9-cis-RA and BMS493 were dissolved in ethanol and used at 1 × 10^-6 ^M unless otherwise indicated. TRAIL (Tebu), TNFα (R&D, Minneapolis, Minnesota), antiFAS antibody (Tebu), Doxorubicin (Tebu), camptothecin (Sigma) and etoposide (Sigma) were used according to the supplier's instructions.

### Measurement of apoptosis

Sub G1 cell-population was quantified by single staining (propidium iodide, PI) according to standard procedures. Briefly, the cells were trypsinized and 2,5 × 10^5 ^cells were washed with PBS 1× and incubated overnight at 4°C in a hypotonic buffer containing propidium iodide (0,1% Triton X-100, 0,1% sodium citrate and 50 μg/ml propidium iodide). DNA fragmentation assays were performed using the Cell Death Detection Elisa kit following the manufacturer's recommendations (Roche). This kit measures the enrichment of histone complexed DNA fragments (mono- and oligonucleosomes) in the cytoplasm of apoptotic cells.

### Oil Red O staining

Cells, grown in coverslips, were fixed with cold 10% Formalin Calcium Acetate for 30 min. After fixation, coverslips were transferred to 60% isopropanol for 1-2 minutes at room temperature (RT). Cells were stained with freshly filtered Oil Red O for 20 min at RT and washed in running water to remove the excess of the staining solution, followed by counterstaining with hematoxylin. The coverslips were then mounted in glycerin jelly.

### RNase protection assays

Total RNA was extracted with Trizol (Gibco BRL). RNase protection assays were performed according to the supplier's instructions (Pharmingen). Routinely, 4 μg total RNA and 6-8 × 10^5 ^cpm of (α-^32^P) uridine triphosphate probe sets were used and after RNase treatment, protected probes were resolved on 5% urea-polyacrylamide-bis-acrylamide gels.

### Subcellular fractionation

T47D cells (3 × 10^6 ^cells per condition) and H3396 cells (2 × 10^6 ^cells) were collected in PBS, centrifuged and resuspended in 200 μl of ice-cold fractionation buffer (0.025% digitonin, 250 mM sucrose, 5 mM Mg Cl2, 10 mM KCl, 1 mM EDTA, 1 mM EGTA, 1 mM PMSF, 20 mM HEPES, pH 7.4 and protease inhibitors) and incubated on ice for 10 minutes. Cell permeabilization was determined by Trypan-blue staining. Cells were then centrifuged at 13,000 rpm and 4°C for 2 minutes. The supernatant containing the cytoplasmic fraction was then isolated from the pellet containing the mitochondrial fraction. The pellet was resuspended in 200 μl RIPA buffer containing 150 mM NaCl, 1% NP-40, 0.5% deoxycholate, 0.1% SDS, 50 mM Tris HCl, pH8 and incubated for 30 minutes on ice. Samples were centrifuged for 10 minutes at 13,000 rpm and 4°C. Release of mitochondrial proteins to the cytosol was assessed by SDS-PAGE gels and Western blotting.

### Measurement of mitochondrial membrane potential

Mitochondrial membrane potential was measured by using the fluorescent dye DiOC6 (3, 30 dihexyloxacarbocyanine iodide) (Sigma) according to the manufacturer's instructions. Briefly, after treatment with retinoids, cells were incubated with 50 nM of DiOC6 at 37°C during 30 minutes. Cells were then washed with PBS and trypsinized. Cells were centrifuged, washed twice with PBS, resuspended in PBS containing 2 μg/ml of propidium iodide and analyzed by FACS.

### Western blotting

Caspase -3, -8, -9, cleaved PARP (Cell Signaling), anti-SMAC (BD Pharmingen), anti-cytochrome c (BD PharMingen) and β-actin (Santa Cruz) antibodies were used to probe blots of extracts prepared using RIPA buffer (20 mM Tris, pH 7.5, 25 mM β-glycerophosphate, 2 mM EDTA, 140 mM NaCl, 1% Triton X-100, 0.1% SDS, 1% sodium deoxycholate, 1 mM PMSF, 1 mM sodium orthovanadate, 2 mM sodium pyrophosphate and protease inhibitors cocktail). cIAP2 (R&D Systems, batch AF817 and AF8171) antibodies were used to probe blots of extracts prepared using Triton Lysis buffer (TLB; 10 mM Tris, pH 7.4, 137 mM NaCl, 2 mM EDTA, 1% Triton X-100, 25 mM β-glycerophosphate, 1 mM sodium orthovanadate, 2 mM sodium pyrophosphate, 10% glycerol, 1 mM PMSF, and protease inhibitors cocktail). Immune complexes were detected by chemiluminescence (Amersham).

### Plasmids

A 1.4 kb fragment corresponding to the 5'-flanking region of cIAP2 gene was amplified by PCR from human genomic DNA and cloned in *XhoI *and *NcoI *of the basic luciferase reporter plasmid pGL3-Luc. A series of 5'deletions of this fragment were amplified by PCR using different forward primers containing a *XhoI *site at their 5' -end and a common reverse primer containing a *NcoI *site at its 5'-end. PCR-amplified DNA fragments were digested with *XhoI *and *NcoI *restriction enzymes, gel purified and inserted into the respective sites in pGL3-Luc vector. Site-directed mutagenesis of the cIAP2 promoter was performed using QuickChange Site-Directed Mutagenesis kit (Stratagene) following manufacturer instructions. Nucleotide sequences were determined by automatic DNA sequencer. Information about primer sequences is available upon request. pSG5-IκBαSR plasmid was constructed by inserting the human cDNA coding for a constitutively activated form of IκBα containing S32A and S36A mutations from the retroviral plasmid pLxSN-IκBαSR(S32A/S36A) into *EcoRI *sites of pSG5 [71].

### Transfection and luciferase assays

Transfections were performed using FuGENE transfection reagent (Roche) following manufacturer instructions. Briefly, 100 ng of luciferase reporter plasmid were transfected along with 30 ng of pCMV-β-galactoside and 100 ng of different expression vectors in 60-70% confluent cells seeded in 24-well plates. 16 hours after transfection, cells were treated for 24 h with 9 cis-retinoic acid at the indicated concentrations. Lysates from transfected cells were analyzed for luciferase and β-galactosidase activity, and data from luciferase activity were normalized by β-galactosidase activity values.

### Electrophoretic mobility shift assays

Nuclear extracts of T47D cells treated with 1 μM 9-cis-RA acid were prepared as described by Dignam et al. [72]. Oligonucleotide probes corresponding to the NF-κB binding site-1 (sense, 5'-ATGGAAATCCCCGA-3' and antisense, 5'-TCGGGGATTTCCAT-3') and NF-κB binding site-3 (sense, 5'-GCTGGAGTTCCCCT-3' and antisense, 5'-AGGGGAACTCCAGC-3') were radiolabeled using T4 polynucleotide kinase in the presence of (γ-32 P) ATP. Radiolabeled double strand oligonucleotides were mixed with 10 μg of nuclear protein extracts in a final volume of 20 μl of binding buffer (20 mM HEPES, pH 7.9, 60 mM KCl, 1 mM Mg Cl_2_, 20 mM EDTA, 0.5 mM dithiothreitol, 10% glycerol) containing 2 μg poly (dI.dC). After 30 minutes of incubation at room temperature, binding complexes were separated on a 5% non-denaturating polyacrylamide gel with 0.5× TBE buffer. The gel was vacuum dried and subjected to autoradiography. For supershift experiments, 0.2 μg of p65 antibody (Santa Cruz) was added to the samples before addition of the radiolabeled oligonucleotide.

### RNA interference

T47D breast cancer cells were seeded 24 h prior to transfection with 100 nM siGENOME SMARTpool for cIAP2 (Dharmacon, Thermo) using DharmaFECT-1 as transfection reagent according to manufacturer's instructions. After 16 h, siRNA-lipid complexes were removed and cells were treated with 9-cis-RA for 30 h prior to etoposide treatment.

### Chromatin Immunoprecipitation

T47D breast cancer cells growing in p150 dishes were treated with 1 μM 9-*cis*-RA for 48 h. Media and ligands were renewed 45 min before chromatin extracts were prepared. ChIP assays were performed according to a previously described procedure [73,74]. Sonication was performed using a Bioruptor UCD-200TM from Diagenode (20 min, high intensity, 30 second on/off interval). Chromatin complexes were incubated with primary rabbit polyclonal antibodies to acetylated H3 histone (06-599, Upstate), RelA/p65 (06-418, Upstate), RAR (M-454, sc-773X), RXRα (D-20, sc553), c-jun (H79, sc-1694X) or normal rabbit serum immunoglobulins (sc-2027). Eluted DNA from the ChIP assays were assayed directly by real-time PCR. DNA inputs were diluted 1:100 previous to real time PCR assay. 1 μl of template was used per 25 μl reaction, all samples were analysed in duplicate using SYBR-green 2× PCR Master Mix (Roche) on a Stratagene Mx3005P real-time PCR thermal cycler. After an initial denaturation and activation incubation of 10 min, 45 cycles of 2-step cycling were performed with an annealing temperature of 60°C with the following primers: forward 5'-AAAGTGTATGGCGGATGGAGG-3' and reverse 5'CGGCATTTACTGAAAGACATTTGC-3' to amplify the cIAP2 promoter region -364/-218; forward 5'-CTCTCTGGCTGTCTGCTTTTGC-3' and reverse 5'-GTGAACTTTCGGTGAACCCTACC-3' to amplify the RARE-containing RARβ2 promoter region (-166/-35); and forward 5'-GCAGCGGAGCATTACCTCATC-3' and reverse primer 5'-CAGTCAACCCCTAAAAATAGCCC-3' to amplify the cJUN promoter region containing the AP1 site (-130/+31). Melting curves were performed to verify product specificity. Relative fold induction over IgG for each immunoprecipitate was assessed by analysing the change in threshold cycle number (delta Ct) upon normalization to their respective inputs.

### Reverse-Transcriptase Polymerase Reaction

Total RNA was isolated using Tri-Reagent (Sigma) and 1 μg of RNA was used in a reverse transcription reaction as instructed using iScript cDNA synthesis kit from Bio-RAD. Quantitative PCR was performed using equal amounts of cDNA with the following primers: cIAP2 mRNA 5'-AGCTGAAGCTGTGTTATATGAGC-3' (forward) and 5'-ACTGTACCCTTGATTGTACTCCT-3' (reverse) and β-actin mRNA 5'-AACTCCATCATGAAGTGTGACG-3' (forward) 5'-GATCCACATCTGCTGGAAGG-3' (reverse).

### Statistical analysis

Student's *t *test was performed using the Microsoft Excell software (version 11.5.4). The statistical significance of difference between groups was expressed by asterisks (*, 0.01 <*P *< 0.05; **, 0.001 <*P *< 0.01; ***, *P *< 0.001).

## Competing interests

AMJ-L and AA declare that they have no competing interests. HG received grant support from the Ligue National Contre le Cancer, the Association for International Cancer Research and the European Community, as specified in the Acknowledgements.

## Authors' contributions

AMJ-L carried out the acquisition, analysis and interpretation of data, wrote the manuscript and contributed to the design and conception of the study. AA contributed to the conception of the study and helped draft the manuscript. HG conceived the studies and helped draft the manuscript. All authors read and approved the paper.

## Supplementary Material

Additional file 1 **9-cis-RA pretreatment prevents apoptosis induced by etoposide in ZR-75-1 breast cancer cells**. ZR-75-1 cells were pretreated with or without 9-cis-RA for 30 h, followed by treatment with different doses of etoposide for 72 h. Apoptotic cells were determined by FACS analysis after staining with propidium iodide as described in "Materials and Methods". Click here for file
