# Supplementary figures and images for: Retinoic acid protects human breast cancer cells against etoposide-induced apoptosis by NF-kappaB-dependent but cIAP2-independent mechanisms
Source: Mol Cancer. 2010 Jan 26;9:15. doi: 10.1186/1476-4598-9-15 (PMC2825243; doi:10.1186/1476-4598-9-15)

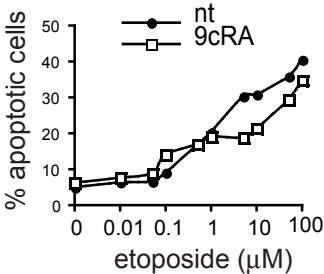

Supplement: Additional file 1 — 9-cis-RA pretreatment prevents apoptosis induced by etoposide in ZR-75-1 breast cancer cells. ZR-75-1 cells were pretreated with or without 9-cis-RA for 30 h, followed by treatment with different doses of etoposide for 72 h. Apoptotic cells were determined by FACS analysis after staining with propidium iodide as described in "Materials and Methods". [file 1476-4598-9-15-S1.PDF]
